# Multidirectional chromosome painting in *Synallaxis frontalis* (Passeriformes, Furnariidae) reveals high chromosomal reorganization, involving fissions and inversions

**DOI:** 10.3897/CompCytogen.v12i1.22344

**Published:** 2018-03-13

**Authors:** Rafael Kretschmer, Vanusa Lilian Camargo de Lima, Marcelo Santos de Souza, Alice Lemos Costa, Patricia C. M. O’Brien, Malcolm A. Ferguson-Smith, Edivaldo Herculano Corrêa de Oliveira, Ricardo José Gunski, Analía Del Valle Garnero

**Affiliations:** 1 Programa de Pós-graduação em Genética e Biologia Molecular, PPGBM, Universidade Federal do Rio Grande do Sul, Porto Alegre, Rio Grande do Sul, RS, Brazil; 2 Programa de Pós-graduação em Ciências Biológicas, PPCGCB, Universidade Federal do Pampa, São Gabriel, Rio Grande do Sul, Brazil; 3 Graduação em Ciências Biológicas, Universidade Federal do Pampa, São Gabriel, Rio Grande do Sul, Brazil; 4 Cambridge Resource Centre for Comparative Genomics, Department of Veterinary Medicine, University of Cambridge, Madingley Road, Cambridge CB3 0ES, UK; 5 Faculdade de Ciências Naturais, Instituto de Ciências Exatas e Naturais, Universidade Federal do Pará, Belém, Pará, Brazil; 6 Laboratório de Cultura de Tecidos e Citogenética, SAMAM, Instituto Evandro Chagas, Ananindeua, Brazil

**Keywords:** Avian cytogenetics, chromosome painting, macrochromosome syntenies, chromosome fission, intrachromosomal rearrangements

## Abstract

In this work we performed comparative chromosome painting using probes from *Gallus gallus* (GGA) Linnaeus, 1758 and *Leucopternis albicollis* (LAL) Latham, 1790 in *Synallaxis frontalis* Pelzeln, 1859 (Passeriformes, Furnariidae), an exclusively Neotropical species, in order to analyze whether the complex pattern of intrachromosomal rearrangements (paracentric and pericentric inversions) proposed for Oscines and Suboscines is shared with more basal species. *S. frontalis* has 82 chromosomes, similar to most Avian species, with a large number of microchromosomes and a few pairs of macrochromosomes. We found polymorphisms in pairs 1 and 3, where homologues were submetacentric and acrocentric. Hybridization of GGA probes showed syntenies in the majority of ancestral macrochromosomes, except for GGA1 and GGA2, which hybridized to more than one pair of chromosomes each. LAL probes confirmed the occurrence of intrachromosomal rearrangements in the chromosomes corresponding to GGA1q, as previously proposed for species from the order Passeriformes. In addition, LAL probes suggest that pericentric inversions or centromere repositioning were responsible for variations in the morphology of the heteromorphic pairs 1 and 3. Altogether, the analysis of our data on chromosome painting and the data published in other Passeriformes highlights chromosomal changes that have occurred during the evolution of Passeriformes.

## Introduction

Passeriformes (passerines) are the largest and most diverse order of birds, with approximately 5,700 species, representing almost 60 % of all living birds (Ericson et al. 2014). The order is divided into two suborders: Oscines (songbirds), which comprise 776 genera and approximately 80 % of all species of Passeriformes, and Suboscines (vocal non-learners), with 284 genera (Selvatti et al. 2015). Among the Suboscines, the family Furnariidae (ovenbirds and woodcreepers) is outstanding for its exceptional diversification and ecological adaptation (Chesser et al. 2004, Moyle et al. 2009). Among its three subfamilies, Furnariinae is the richest in number of species (Irestedt et al. 2002, Remsen et al. 2016).

Among birds, Passeriformes have the highest number of species analyzed by classical cytogenetics (Santos and Gunski 2006). Most species show diploid numbers (2n) ranging between 76–80 chromosomes, although there are exceptions, such as *Platyrinchus mystaceus* Vieillot, 1818 a Suboscine species belonging to the Platyrinchidae family, which has 60 chromosomes (Gunski et al. 2000, Santos and Gunski 2006, Correia et al. 2009). Among the Furnariidae, only two species have been described cytogenetically - *Sittasomus griseicapillus* Vieillot, 1818 and *Lepidocolaptes angustirostris* Vieillot, 1818, both with 2n=82 (Barbosa et al. 2013).

Besides information on diploid number and chromosome morphology, classical cytogenetic analyses have detected examples of chromosomal polymorphisms in some species of Passeriformes, such as *Saltator similis* d’Orbigny and Lafresnaye, 1837 (dos Santos et al. 2015) and *Zonotrichia albicollis* Gmelin, 1789 (Thomas et al. 2008). The polymorphism found in the latter species was associated with plumage and behavioral variations (Thomas et al. 2008), corroborating the fact that chromosomal alterations may have important effects on genome function, aside from being important phylogenetic markers.

Fourteen species of the suborder Oscines have been analyzed by chromosome painting (Guttenbach et al. 2003, Derjusheva et al. 2004, Itoh and Arnold 2005, Nanda et al. 2011, Kretschmer et al. 2014, dos Santos et al. 2015, 2017), and only four species of Suboscines (Kretschmer et al. 2015, Rodrigues et al. In press). Eight of these species were compared using only whole chromosome probes of *Gallus gallus* Linnaeus, 1758 (GGA) (Guttenbach et al. 2003, Derjusheva et al. 2004, Itoh and Arnold 2005, Nanda et al. 2011). The results have shown mostly the same syntenic groups found in the putative avian ancestral karyotype (PAK) proposed by Griffin et al. (2007), with the exception of PAK1 (GGA1), which is split into two chromosome pairs, representing a synapomorphy shared by all the species of Passeriformes analyzed so far. However, the other ten species were analyzed with whole chromosome probes from two different species – GGA and *Leucopternis albicollis* Latham, 1790 (LAL), an Accipitridae with 2n=66, in which syntenic groups corresponding to PAK pairs 1, 2, 3 and 5 each correspond to 2–5 different pairs (de Oliveira et al. 2010, Kretschmer et al. 2014, 2015, dos Santos et al. 2015, 2017, Rodrigues et al. In press). This approach revealed additional rearrangements in which chromosome pairs corresponding to PAK1p and PAK1q were reshuffled through a series of paracentric and pericentric inversions in both Oscines and Suboscines species, suggesting that these rearrangements had occurred early in the history of Passeriformes, before their split into two suborders (Kretschmer et al. 2014, 2015, dos Santos et al. 2015, 2017). The occurrence of such intrachromosomal rearrangements has been confirmed by data from genome sequencing in Passeriformes (Frankl-Vilches et al. 2015).

Species belonging to the genus *Synallaxis* Vieillot, 1818 (Subfamily Furnariinae) show higher diversification when compared to other Furnariidae, probably due to the shift in their nesting habits and an expansion of their habitats to open areas (Irested et al. 2009), but only a few species of this family have been karyotyped. Hence, the aim of this study was to analyze the karyotype of *Synallaxis frontalis* Pelzeln, 1859, a species belonging to family Furnariidae, by chromosome painting using GGA and LAL probes, in order to verify if this complex pattern of intrachromosomal rearrangements is also present in more basal species for Passeriformes.

## Material and methods

### Samples and chromosome preparations

The experiments followed protocols approved by the ethics committee (CEUA-Universidade Federal do Pampa, no. 026/2012, SISBIO 33860-3 and 44173-1). Seven specimens of *Synallaxis frontalis* (SFR), four males and three females, were caught in São Gabriel, Rio Grande do Sul State, Brazil, within the natural area of Universidade Federal do Pampa. Skin biopsies were used for fibroblast cultures according to Sasaki et al. (1968) and chromosomes were obtained by standard protocols using colcemid and fixation with Carnoy fixative.

### Classical cytogenetics

Diploid number and chromosome morphology were determined by the analysis of at least 20 metaphases per individual, conventionally stained with Giemsa. C-banding (Ledesma et al. 2002) was used to analyze the distribution of blocks of constitutive heterochromatin. Chromosomes were ordered following size and centromere position, according to Ladjali-Mohammedi et al. (1999).

### Fluorescent *in situ* hybridization

Fluorescent *in situ* hybridization (FISH) experiments were performed with whole chromosome probes from two different species – *Gallus gallus* (pairs GGA1-GGA10) and *Leucopternis albicollis* (LAL), pairs homologous to GGA 1 (LAL 3, 6, 7, 15 and 18), GGA 2 (LAL 2, 4 and 20), GGA 3 (LAL 9, 13, 17 and 26), GGA 4 (LAL 1 and 16), GGA 5 (LAL 5) and GGA 6 (LAL 3) (de Oliveira et al. 2010). Both sets of probes were obtained by flow cytometry at the Cambridge Resource Centre for Comparative Genomics (Cambridge, United Kingdom), and labeled by DOP-PCR, using biotinilated nucleotides. Hybridization, stringency washes and detection were performed according to de Oliveira et al. (2010). Slides were analyzed using a fluorescence microscope (Zeiss Imager Z2) and images were captured using the software Axiovision 4.8 (Zeiss, Germany).

## Results

### Classical cytogenetics

**Figure 1.**
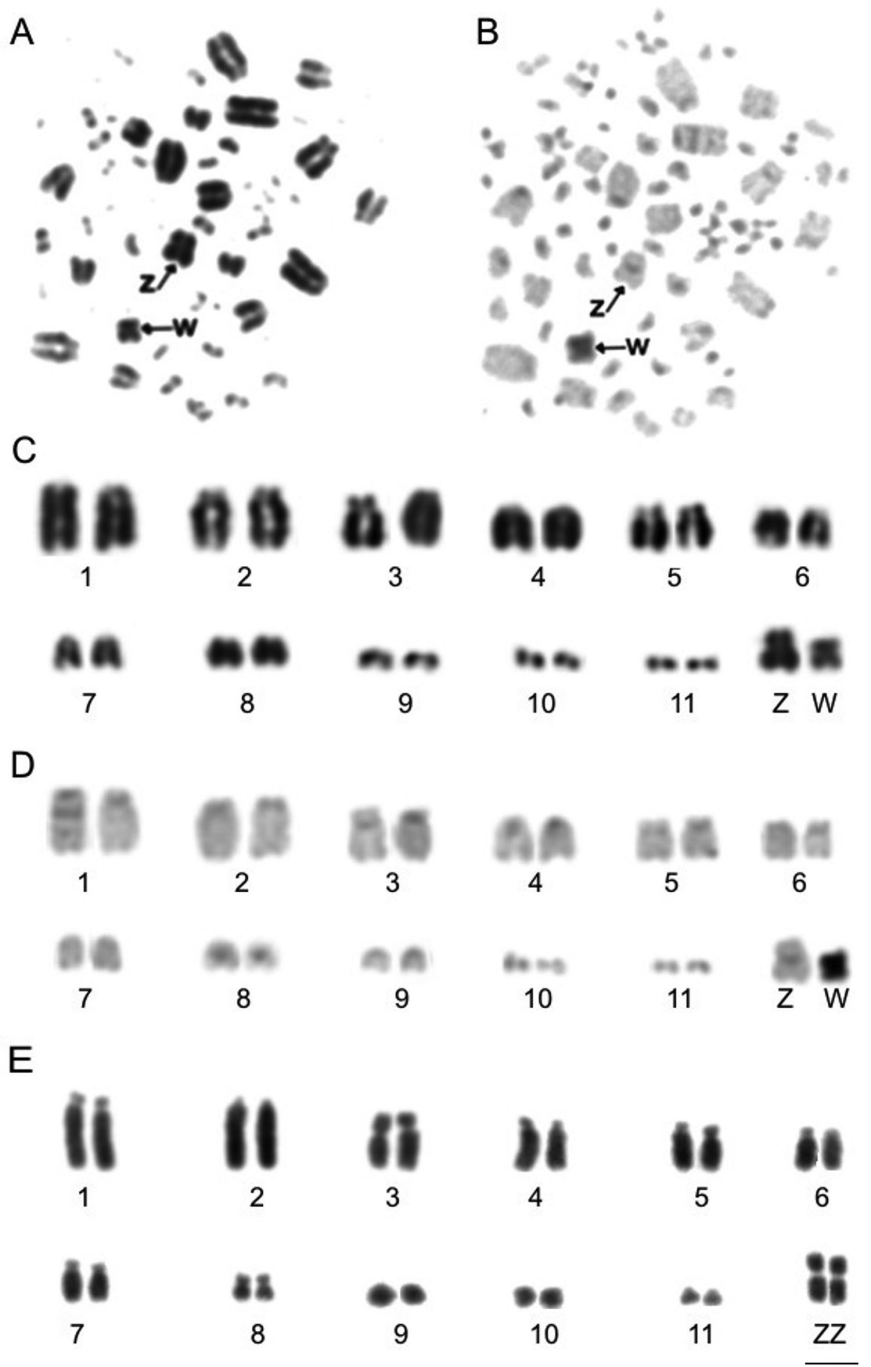
Metaphases and partial karyotype of female *Synallaxis frontalis* with heteromorphism in pair 1: Giemsa (**A, C**), C-banding (**B, D**). Partial karyotype of male *S. frontalis* with heteromorphism in pair 3 (**E**). Arrows indicate the Z and W chromosomes. Scale bar: 5 µm.

We found a karyotype of 2n=82 in *Synallaxis frontalis*, with 11 pairs of macrochromosomes, including the Z and the W chromosomes, and 30 pairs of microchromosomes (Figure 1A–B). In some individuals, pairs 1 and 3 showed heteromorphism of the length of their short arms (Figure 1). Hence, pair 1 was represented by a submetacentric and an acrocentric element in one male and one female, while in the other five individuals both homologues of this pair were acrocentric. Additionally, chromosomes of pair 3 were heteromorphic (acrocentric and submetacentric) in two males and one female, while in the other four individuals this pair was acrocentric. In two individuals, pairs 1 and 3 were both acrocentric. Pair 2 and 4–7 were acrocentric, while pair 8 was metacentric and the Z chromosome was submetacentric in all individuals. The W chromosome was metacentric (Fig. 1A–D).

C-banding showed that blocks of constitutive heterochromatin were located in the centromeric region of the autosomes and Z chromosome, while the W chromosome was almost completely heterochromatic (Figure 1B, D).

**Figure 2.**
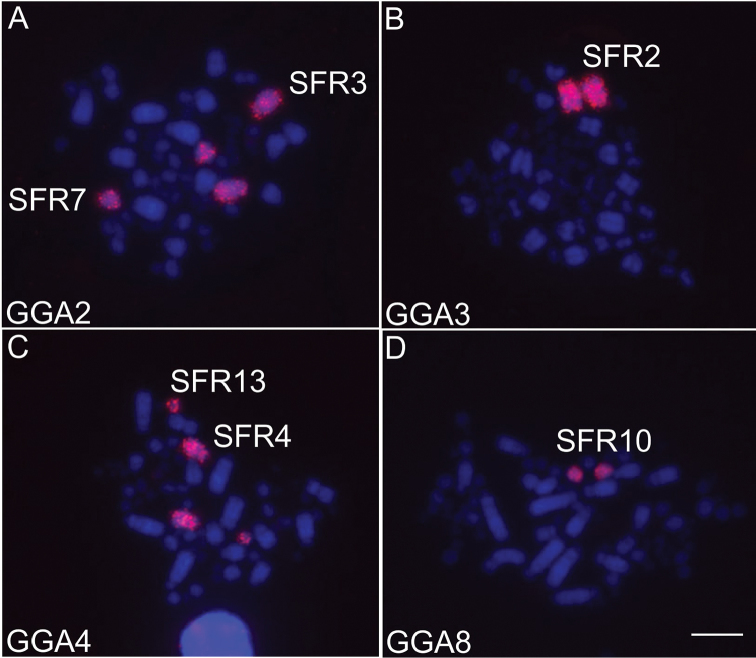
Representative FISH experiments using *Gallus gallus* GGA (**A–D**) probes on metaphase chromosomes of *Synallaxis frontalis* (SFR). Chromosomes were counterstained with DAPI (blue), and probes detected with Cy3 (red). Probes used are indicated in the lower left corner of the images. Scale bar: 5 µm.

**Figure 3.**
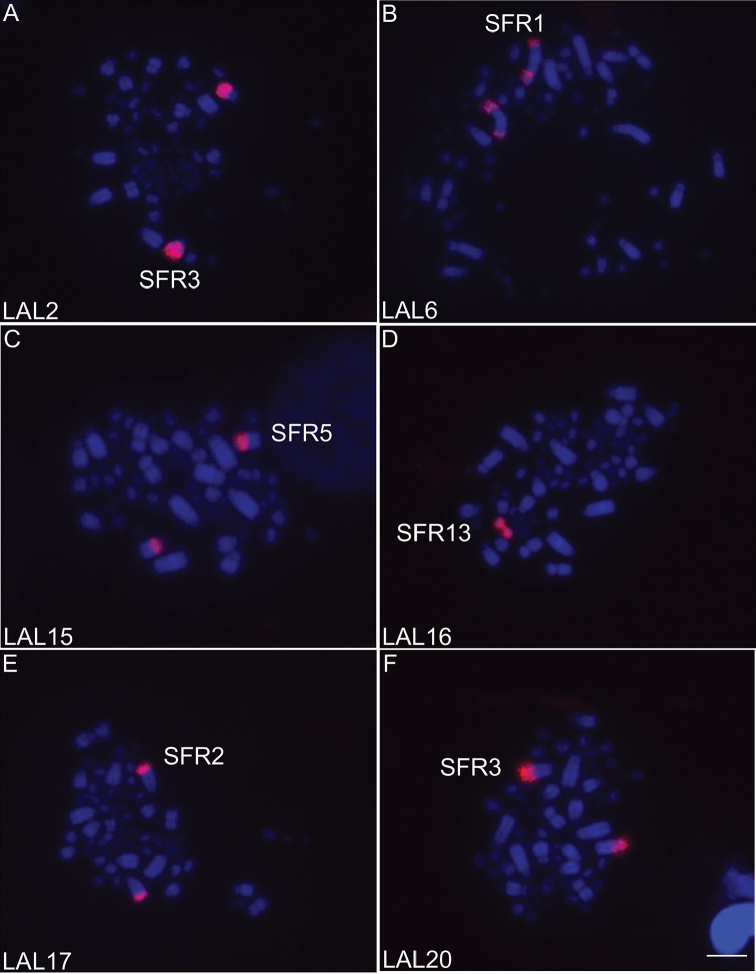
Representative FISH experiments using *Leucopternis albicollis* LAL (**A–F**) probes on metaphase chromosomes of *Synallaxis frontalis* (SFR). Chromosomes were counterstained with DAPI (blue), and probes detected with Cy3 (red). Probes used are indicated in the lower left corner of the images. Scale bar: 5 µm.

**Figure 4.**
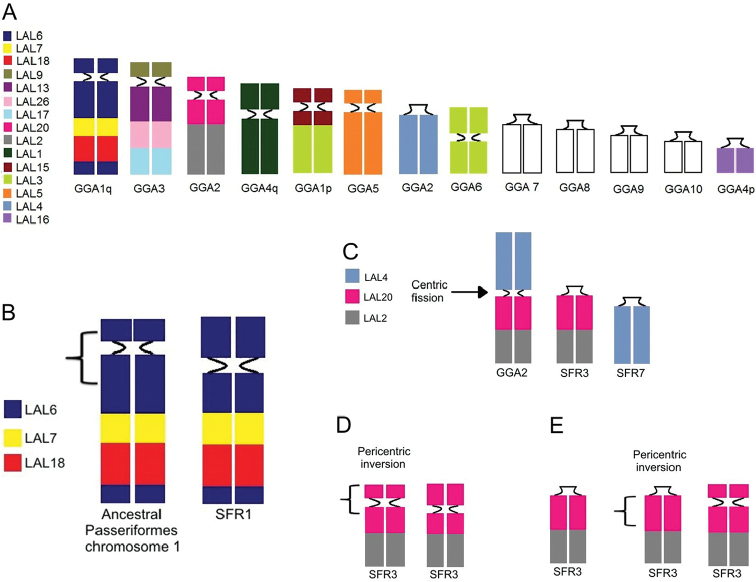
Homology map (13 first autosomal pairs) comparing the syntenic groups of *Synallaxis frontalis* to *Gallus gallus* (bottom) and *Leucopternis albicollis* (colors) (**A**). Schematic diagram showing the hypothetical pericentric inversion responsible for the heteromorphism observed in pair 1 from two individuals of *Synallaxis frontalis* (SFR1) (**B**). Hypothetical rearrangements observed in *Synallaxis frontalis* (SFR) PAK 2 (GGA2) that would have given rise to SFR3 and SFR7 (C–E). First, a centric fission in the ancestral synteny homologous to GGA2, created two distinct chromosome pairs, homologous to GGA2p (SFR7) and GGA2q (SFR3) (**C**). A pericentric inversion in SFR3 changed its morphology to acrocentric (**D**). A second pericentric inversion gave rise to the heteromorphic element in pair 3, which corresponds to a submetacentric chromosome (**E**).

GGA whole chromosome probes showed that most syntenic groups found in the putative avian ancestral karyotype (PAK) were conserved in SFR, except for GGA 1 and GGA 2, which were fissioned into two pairs each - SFR1/SFR5 and SFR3/SFR7, respectively (Fig. 2). LAL probes confirmed that both fissions were centric (Fig. 3). The results also suggested that pericentric inversions or centromere repositioning were responsible for the heteromorphism observed in pairs SFR1 and SFR3 (figs 5, 6). Moreover, the complex pattern of intrachromosomal rearrangements involving paracentric and pericentric inversions previously described in other Passeriformes in the chromosome corresponding to GGA1q were also detected by LAL probes. The homology map comparing SFR, GGA and LAL chromosomes is shown in Figure 4A.

## Discussion

The genome of *S. frontalis* shows a chromosomal organization typical for Class Aves and order Passeriformes (Gunski et al. 2000, Santos and Gunski 2006, Kretschmer et al. 2014), with 2n=82. The two species of the family Furnariidae described cytogenetically so far, *Sittasomus griseicapillus* and *Lepidocolaptes angustirostris*, also have the same diploid number found in *S. frontalis*, but with variations in the morphologies of some macrochromosomes (Barbosa et al. 2013). The Z chromosome of *S. frontalis* is submetacentric, unlike the acrocentric morphology found in *S. griseicapillus*, and *L. angustirostris*. The variation in morphology of this chromosome is common, even in species of the same family, as observed in family Tyrannidae (Gunski et al. 2000, Kretschmer et al. 2015), probably due to the presence of repetitive sequences in this chromosome (Nanda et al. 2002). Similarly to these two species, the W chromosome is metacentric. Concerning heteromorphic pairs 1 and 3, it is interesting to notice that heteromorphisms in autosomal chromosomes are not exclusive to *S. frontalis*, since they have been reported in species of the Emberizidae family (genera *Zonotrichia* Swainson, 1832 and *Junco* Wagler, 1831), as well as in the Z chromosome of *Saltator similis* (Thraupidae) (Shields 1973, de Lucca 1985, Thomas et al. 2008, dos Santos et al. 2015). Additionally, the C-banding pattern in *S. frontalis* is similar to most bird species, with blocks of constitutive heterochromatin in the centromeric region of chromosomes and in most of the W chromosome (Kretschmer et al. 2014, dos Santos et al. 2015).

Most of the ancestral macrochromosomes are conserved as whole chromosomes in *S. frontalis*, as shown by the hybridizations of *G. gallus* macrochromosomes. Only the first two pairs (GGA1 and GGA2) are not conserved, due to the occurrence of fissions, and correspond to SFR1 and SFR5, SFR3 and SFR7 pairs, respectively. The fission of the ancestral chromosome 1 has been found in all species of the order Passeriformes studied to date (19 species, including *S. frontalis*) (Guttenbach et al. 2003, Derjusheva et al. 2004, Itoh and Arnold 2005, Nanda et al. 2011, Kretschmer et al. 2014, 2015, dos Santos et al. 2015, 2017, Rodrigues et al. In press). Probably this characteristic is shared by all Passeriformes, since it was found in species of both suborders, Oscines (14 species) and Suboscines (5 species, including *S. frontalis*). The presence of this characteristic in the genome of *S. frontalis* is important for the confirmation of this hypothesis, since only four species of the Suboscines suborder had been studied by chromosome painting, and now we can verify that this characteristic is shared between two species in different families, Tyrannidae and Furnariidae (Kretschmer et al. 2015, Rodrigues et al. In press). In addition, the Furnariidae family is more basal than the family Tyrannidae (Selvatti et al. 2015).

Unlike the fission of the GGA1 chromosome, the fission of the GGA2 chromosome has been described previously in only one species of the order Passeriformes, *Satrapa icterophrys* Vieillot, 1818 (Rodrigues et al. In press). This rearrangement is probably shared with two other species of the Furnariidae family described by Barbosa et al. (2013), because in these species the two first autosomes pairs are similar in size, a fact also observed in *S. frontalis*. Two species of the Formicariidae family (Furnariidae sister group) also present the first two pairs with similar size, so the fission of the GGA2 chromosome may be a characteristic shared by the species of Parvordem Furnariida (Ledesma et al. 2002, Selvatti et al. 2015). This similarity was not observed in other Passeriformes analyzed by chromosome painting until now, suggesting the possibility of fission of chromosome 2 in other species of Furnariidae and Formicariidae (Guttenbach et al. 2003, Derjusheva et al. 2004, Itoh and Arnold 2005, Nanda et al. 2011, Kretschmer et al. 2014, 2015, dos Santos et al. 2015, 2017). It is necessary to confirm this hypothesis by chromosome painting in different families of Parvordem Furnariida. In addition, as the fission of chromosome GGA2 has been observed in only one Tyrannidae species up to this moment, this rearrangement corresponds probably to a convergent character in *Satrapa icterophrys* and in Furnariidae species.

Hybridizations with LAL probes was not enough to identify the mechanism responsible for the heteromorphisms observed in the first and third chromosomes pairs in some SFR individuals. Both heteromorphisms may have originated either by pericentric inversions or centromere repositioning. Pericentric and paracentric inversions have been reported in several species of Passeriformes (Warren et al. 2010, Volker et al. 2010, Skinner and Griffin 2012, Kretschmer et al. 2014, 2015, dos Santos et al 2015, 2017). However, an alternative explanation is the centromere repositioning that was also reported in Galliformes species (Kasai et al. 2003, Zlotina et al., 2012). We have assumed that the mechanism was a pericentric inversion, since several *in silico* and chromosome painting studies have demonstrated a high frequency of inversions in bird species, especially Passeriformes (Warren et al. 2010, Volker et al. 2010, Skinner and Griffin 2012, Kretschmer et al. 2014, 2015). Thus, an extra inversion may had occurred in the region corresponding to LAL6 (GGA1q) (Fig. 4B) in one of the homologous chromosomes in individuals with heteromorphisms in the first pair, in addition to the three inversions common to all Passeriformes. Similarly, in individuals with heteromorphisms in the third pair, there was an inversion in the segment corresponding to LAL20 in one homologue (Fig. 4C–D).

In addition to the *in silico* analysis demonstrating several intrachromosomal rearrangements, chromosome painting studies with *L. albicollis* probes have also identified some of these rearrangements, especially in the GGA1q chromosome in Passeriformes (Kretschmer et al. 2014, 2015, dos Santos et al. 2015, 2017). Here, we have identified inversions already proposed for the Passeriformes (GGA1p and q) and hypothetical inversions responsible for the heteromorphisms in the first and third pairs. However, the rearrangements of the chromosome that corresponds to GGA1q detected by chromosome painting with LAL probes is more complex than we imagined initially. In 2014, Kretschmer and colleagues first described three inversions on chromosome two (GGA1q) in two species of the genus *Turdus* Linnaeus, 1758 (Oscines). In 2015, the same three inversions were detected in *Elaenia spectabilis* Pelzeln, 1868 (Suboscines) (Kretschmer et al. 2015). After the publication of the *E. spectabilis* observation, dos Santos et al. (2015) and dos Santos et al. (2017) also detected the reorganization of chromosome 2 (GGA1q) in two species of the genus *Saltator* Vieillot, 1816 (Oscines), *Taeniopygia guttata* Reichenbach, 1862 and in *Serinus canaria* Linnaeus, 1758, but this rearrangement was slightly different from that in *Turdus* and *E. spectabilis*. The main difference is that the block corresponding to LAL 18 is conserved integrally in the four species described by dos Santos (et al. 2015, 2017), whereas in *Turdus* and *E. spectabilis* this region is separated into two blocks. The most likely explanation would be the occurrence of independent rearrangements in Oscines and Suboscines, since a block of LAL 18 appears in *Saltator*, *Taeniopygia guttata*, *Serinus canaria* and *Synallaxis* while two blocks appear in *Turdus* (Oscines) and *Elaenia* (Suboscines). However, we still cannot determine which of these characters was present in the last common ancestor of Passeriformes. Perhaps it was the pattern observed in *S. frontalis*, since it is the most basal species of the order Passeriformes studied to date, but other species of the Furnariidae family must be analyzed to confirm or reject the hypothesis of independent rearrangement. However, the current scenario leads us to assume that the ancestral genome of the Passeriformes had a complex reorganization of the chromosomes corresponding to GGA1q, although it is necessary to determine which of the two situations was the first to occur – the one observed in *S. frontalis*, *Saltator*, *Taeniopygia guttata* and *Serinus canaria* or the one found in *Turdus* and *Elaenia*.

Future studies on this species could use other probes such as BACs clones (Damas et al. 2017) to test if the heteromorphisms described here were originated by pericentric inversions or centromere repositioning. Besides that, it would be interesting to carry out similar work to the present study in the sympatric sister species *S. spixi*, since Hooper and Price (2017) proposed that chromosomal inversion differences correlate with range overlap in passerine birds. The conventional analysis with Giemsa in other individuals of *S. frontalis* would also be useful in order to verify if these heteromorphisms are fixed in the population sampled and in other populations. In addition, it would be interesting to analyze the possible effects of these heteromorphisms on the phenotype of carriers, since in *Zonotrichia albicollis* it has been proposed that the heteromorphisms caused changes in behavior and plumage (Thomas et al. 2008).
